# The Etiological Role of Common Respiratory Viruses in Acute Respiratory Infections in Older Adults: A Systematic Review and Meta-analysis

**DOI:** 10.1093/infdis/jiy662

**Published:** 2019-03-08

**Authors:** Ting Shi, Andrew Arnott, Indre Semogas, Ann R Falsey, Peter Openshaw, Jadwiga A Wedzicha, Harry Campbell, Harish Nair, The RESCEU Investigators, Harish Nair, Harry Campbell, Ting Shi, Shanshan Zhang, You Li, Peter Openshaw, Jadwicha Wedzicha, Ann Falsey, Mark Miller, Philippe Beutels, Louis Bont, Andrew Pollard, Eva Molero, Federico Martinon-Torres, Terho Heikkinen, Adam Meijer, Thea Kølsen Fischer, Maarten van den Berge, Carlo Giaquinto, Rafael Mikolajczyk, Judy Hackett, Eskinder Tafesse, Bing Cai, Charles Knirsch, Antonio Gonzalez Lopez, Ilse Dieussaert, Nadia Dermateau, Sonia Stoszek, Scott Gallichan, Alexia Kieffer, Clarisse Demont, Arnaud Cheret, Sandra Gavart, Jeroen Aerssens, Veronique Wyffels, Matthias Cleenewerck, Robert Fuentes, Brian Rosen

**Affiliations:** 1 Centre for Global Health Research, Usher Institute of Population Health Sciences and Informatics, University of Edinburgh; 2 National Heart and Lung Institute, Imperial College London, United Kingdom; 3 University of Rochester School of Medicine, New York; 4 ReSViNET Foundation, Zeist, The Netherlands

**Keywords:** Etiological role, respiratory virus, acute respiratory infection, older adults

## Abstract

Acute respiratory tract infections (ARI) constitute a substantial disease burden in adults and elderly individuals. We aimed to identify all case-control studies investigating the potential role of respiratory viruses in the etiology of ARI in older adults aged ≥65 years. We conducted a systematic literature review (across 7 databases) of case-control studies published from 1996 to 2017 that investigated the viral profile of older adults with and those without ARI. We then computed a pooled odds ratio (OR) with a 95% confidence interval and virus-specific attributable fraction among the exposed (AFE) for 8 common viruses: respiratory syncytial virus (RSV), influenza virus (Flu), parainfluenza virus (PIV), human metapneumovirus (HMPV), adenovirus (AdV), rhinovirus (RV), bocavirus (BoV), and coronavirus (CoV). From the 16 studies included, there was strong evidence of possible causal attribution for RSV (OR, 8.5 [95% CI, 3.9–18.5]; AFE, 88%), Flu (OR, 8.3 [95% CI, 4.4–15.9]; AFE, 88%), PIV (OR, not available; AFE, approximately 100%), HMPV (OR, 9.8 [95% CI, 2.3–41.0]; AFE, 90%), AdV (OR, not available; AFE, approximately 100%), RV (OR, 7.1 [95% CI, 3.7–13.6]; AFE, 86%) and CoV (OR, 2.8 [95% CI, 2.0–4.1]; AFE, 65%) in older adults presenting with ARI, compared with those without respiratory symptoms (ie, asymptomatic individuals) or healthy older adults. However, there was no significant difference in the detection of BoV in cases and controls. This review supports RSV, Flu, PIV, HMPV, AdV, RV, and CoV as important causes of ARI in older adults and provides quantitative estimates of the absolute proportion of virus-associated ARI cases to which a viral cause can be attributed. Disease burden estimates should take into account the appropriate AFE estimates (for older adults) that we report.

## Teaser

The etiological roles of common respiratory viruses in acute respiratory infections in older adults are important and should be taken into consideration in disease burden estimates.

Acute respiratory tract infections (ARI), including pneumonia, constitute a substantial disease burden in adults and elderly individuals. Respiratory viruses are detected more frequently than bacteria in adults with pneumonia [1]. The substantial contribution of viruses to ARI hospitalizations among adults is being increasingly recognized [2, 3].

Although influenza virus (Flu) is the most widely recognized viral infection associated with respiratory illness, >25 viruses have been linked to pneumonia, causing a substantial disease burden in adults and elderly individuals. These include common pathogens such as rhinovirus (RV), respiratory syncytial virus (RSV), Flu, human metapneumovirus (hMPV), parainfluenza viruses (PIV), and human coronaviruses (CoVs) [2]. RSV is associated with a substantial disease burden in adults, especially among older adults (aged ≥65 years) [4]. Moreover, adults hospitalized with RSV disease can develop severe respiratory complications [5]. RV has been associated with severe respiratory disease outbreaks in adults in long-term care facilities in several settings [6]. Despite advances in diagnostic technology, defining the specific causes of viral pneumonia is challenging, particularly among older adults who may have lower viral loads and for whom viral diagnosis is frequently not considered and/or testing is not performed [7]. Therefore, it is necessary to measure concurrently the background prevalence of nasopharyngeal viral infection in a control (asymptomatic) group, to investigate the etiological role of viruses in older adults with ARI to help inform decisions on prevention and management strategies.

Previously, we have conducted a systematic review to understand the etiological role of common respiratory viruses, focusing on children aged <5 years [8]. To the best of our knowledge, similar estimates for adults are lacking. Therefore, we aimed to conduct a similar systematic review to identify all case-control studies since 1996 investigating the potential role of respiratory viruses in the etiology of ARIs in older adults aged ≥65 years.

## METHODS

### Search Strategy and Selection Criteria

We conducted a systematic review across 7 databases (including 3 Chinese databases) following the approach detailed in the PRISMA (Preferred Reporting Items for Systematic Reviews and Meta-analyses) guidelines [9]. Tailored search strategies were developed and used to search the Medline, Embase, Global Health, LILACS, China National Knowledge Infrastructure (CNKI), Wanfang Data, and Chongqing VIP databases (Appendix). We further searched the reference lists of relevant articles for eligible articles. All searches were limited to between January 1996 and August 2017. No publication status criteria or language restrictions were used. We included studies that fulfilled the following selection criteria (Supplementary Figure 1).

Three investigators (T. S., A. A., and I. S.) conducted independent searches of the English-language literature and extracted data by using standardized data extraction templates. One investigator (T. S.), whose first language is Chinese, searched and extracted data from Chinese-language databases (ie, CNKI, Wanfang, and CQVIP).

The protocol of this review was published in the PROSPERO database (no. CRD42017083332).

### Definitions

The case group was defined as older adults with ARI or pneumonia aged ≥65 years, adapted from World Health Organization Integrated Management of Adolescent and Adult Illness [10]. The details of the definitions are displayed in Supplementary Table 1. The control group was defined as older adults aged ≥65 years who were either healthy or did not have any respiratory symptoms. We categorized countries as either industrialized or developing, on the basis of 2015 criteria from the United Nations Children’s Fund [11].

### Statistical Analysis

We calculated odds ratios (ORs) as the ratio of the odds of detecting each virus in older adults with ARI or pneumonia to the odds of detecting each virus in healthy or asymptomatic controls, with accompanying 95% confidence intervals (CIs). We used a continuity correction of 0.0005 if a virus was detected in one group but not the other [12]. This allowed calculation of an OR for these instances and enabled inclusion in subsequent meta-analyses.

Using Stata (version 13.0), we performed a meta-analysis of virus-specific ORs and reported pooled meta-estimates with corresponding 95% CIs, using the random effects model (ie, the DerSimonian-Laird method) because the included studies are heterogeneous in various aspects and are thus assumed to have different effect sizes [13]. The virus-specific attributable fraction among the exposed (AFE) was used to quantify the etiological role of each virus in patients with ARI. This is an estimate of the percentage of ARIs that can be attributed to each virus, in absolute terms [14], and was calculated as 100 * [OR − 1]/OR, with a 95% CI (from the corresponding 95% CI of the OR). Moreover, for a specific virus, if all included studies did not report any virus detection in one group consistently (usually the control group), we assumed that a strong association indicating a possible causal role for this virus in ARI could be concluded. In these circumstances, we considered that there was no need to run a meta-analysis that would only result in an extremely high OR point estimate and an AFE approaching 100%.

## RESULTS

**Figure 1.**
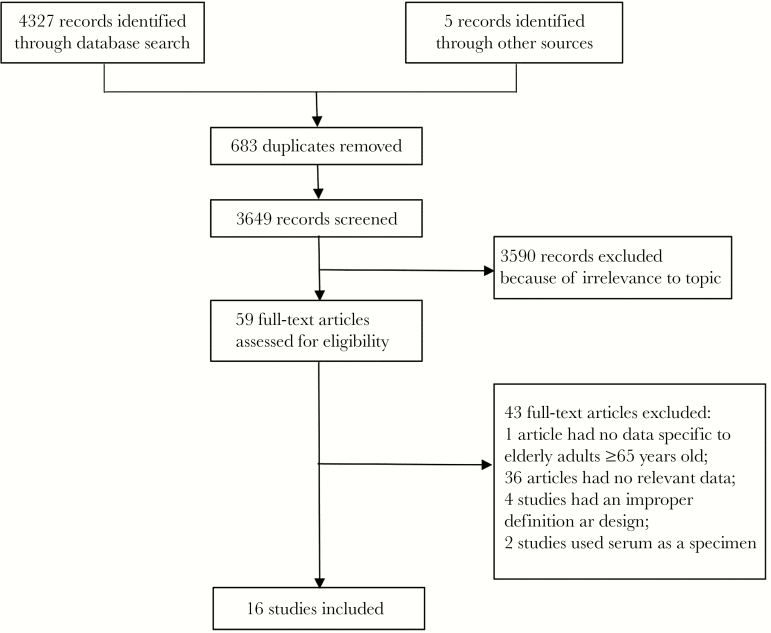
PRISMA (Preferred Reporting Items for Systematic Reviews and Meta-analyses) flow diagram of the literature search.

We identified 4327 (239 from Chinese databases) records through the literature search and 5 records from the reference lists of relevant articles. Among them, only 16 studies (including 2 from Chinese databases) fulfilled our inclusion and exclusion criteria (Figure 1) [1, 15–29]. Forty-three studies were excluded for a variety of reasons: no data specific to older adults ≥65 years old were available (n = 1), the case or control definitions were not fulfilled (n = 4), no applicable data for cases and controls were reported (n = 36), or serum was used as the clinical specimen (n = 2). Seven studies were conducted within developing countries, while 9 were from developed countries (Supplementary Table 2). Although the search was performed for articles published since 1996, all included studies were published since 2003.

All included studies were case-control studies with adults who had ARI or pneumonia in the case group and asymptomatic or healthy adults in the control group. Methods varied among studies. Among the case definitions used, 7 studies used ARI or acute lower respiratory tract infection, while the others used (severe) pneumonia (n = 9). All studies investigated a control group, which had no respiratory symptoms, and in 3 studies healthy older adults (without acute illness) served as controls. Of the case ascertainment methods used, 8 articles recruited the cases from inpatients; 1, from outpatients; 3, from general practices; 1, from the community; and 3, from mixed settings (outpatient settings and the emergency department, and both outpatient and inpatient settings). In 11 studies, controls were ascertained in hospital-based outpatient or clinic sites, whereas in 5 studies, controls were identified from the community. All studies collected a mixture of nasopharyngeal swab specimens, nasopharyngeal aspirates, nasopharyngeal washes, oropharyngeal swab specimens, and nasal/throat swab specimens as the clinical specimen. All studies used polymerase chain reaction analysis (PCR; in some, PCR was combined with serologic analysis or culture) as the diagnostic test.

Meta-analyses of virus-specific ORs are reported as well as the corresponding attributable fractions among the exposed (Supplementary Table 3). RSV, Flu (including Flu A), hMPV, RV, and CoV (also CoV OC43 and 229E) were significantly more common in older adults with a diagnosis of ARI or pneumonia than in asymptomatic or healthy controls (ORs, 8.5 [95% CI, 3.9–18.5], 8.3 [95% CI, 4.4–15.9], 9.8 [95% CI, 2.3–41.0], 7.1 [95% CI, 3.7–13.6], and 2.8 [95% CI, 2.0–4.1], respectively). These viruses had statistically significant positive AFEs, which showed clear associations between these viruses and ARI or pneumonia in older adults. Moreover, PIV (including PIV1 and PIV3; data for PIV2 and PIV4 were not available), Flu B, and AdV were only identified in cases consistently across all included studies (8, 5, and 8 studies, respectively) and absent in control groups. Thus, these viruses were all assumed to have strong associations with ARI. Only 2 studies had data available for BoV, and although both reported virus detection in a greater proportion of cases than controls [18, 28], the association remains a question for further research.

A subgroup analysis was performed to explore the roles of viruses in ARI with respect to region: developing countries and industrialized countries. The meta-estimate OR was higher in industrialized countries as compared to developing countries in the case of Flu (with overlapping 95% CIs), while it was similar for PIV, AdV, and RV. There were insufficient studies to conduct a similar subgroup analysis for other viruses. A sensitivity analysis was performed to investigate the roles of these common viruses in older adults admitted to hospitals with ARI or pneumonia [1, 16, 20, 22, 23, 25, 27, 28]. Eight studies were included, and results are presented in Supplementary Table 4. The meta-estimate OR did not differ significantly from the previous estimate, in which cases from other settings (ie, outpatient and general practice settings) were also included.

## DISCUSSION

This is the first systematic review to evaluate and summarize the literature that includes concurrent control data on the viral etiology of ARI in older adults. Our review summarized data from 5560 cases of ARI in older adults reported across 16 studies. We demonstrated strong evidence in support of a potential causal attribution when a virus is identified in older adults presenting with ARI or pneumonia for RSV (OR, 8.5 [95% CI, 3.9–18.5]; AFE, 88%), Flu (OR, 8.3 [95% CI, 4.4–15.9]; AFE, 88%), PIV (OR, not available [NA]; AFE, approximately 100%), hMPV (OR, 9.8 [95% CI, 2.3–41.0]; AFE, 90%), AdV (OR, NA; AFE, approximately 100%), RV (OR, 7.1 [95% CI, 3.7–13.6]; AFE, 86%), and CoV (OR, 2.8 [95% CI, 2.0–4.1]; AFE, 65%). This supports an etiological role of RSV, Flu, PIV, hMPV, AdV, RV, and CoV in ARI and pneumonia in older adults, thereby indicating a potential for substantive reductions in the number of ARI cases if older adults were vaccinated against these viruses or treated with antivirals. For the other respiratory viruses studied, the role of BoV in ARI and pneumonia was uncertain because of the limited evidence available from the published literature, requiring more research to clarify its role in older adults with ARI. A sensitivity analysis focusing only on older adults who were admitted to hospitals with ARI or pneumonia did not differ significantly from our estimate, in which patients from all settings were considered. This might result from the limited number of studies available to provide a more robust sensitivity analysis. No studies calculated adjusted ORs to account for confounding effects from age or season, which might compromise the actual association and should be considered in the study design in future research.

These findings should inform the results of studies that seek to estimate the global, regional, and national burden of disease due to these viruses in older adults [30]. They show that RSV, Flu, PIV, hMPV, AdV, RV, and CoV are important causes of ARI in older adults, and disease burden estimates should take into account the appropriate AFE estimates (for older adults) that we report, rather than the AFE estimates in children aged <5 years. There is considerable international attention on RSV-associated ARI in older adults at this time, during which novel vaccine and antiviral strategies are being evaluated and prioritized [31, 32], and more-accurate disease burden estimates (using these results) would help to inform future policies and interventions.

The prevalence of virus detection from etiologic studies of pneumonia in adults is substantially lower than the detection rate in studies of children. The EPIC (Etiology of Pneumonia in the Community) study team showed that the viruses were detected in 26% of adults who had been hospitalized with community-acquired pneumonia, compared with 73% of children who were admitted to the hospital [1, 33]. There are several reasons for such low levels of detection, such as the inability to obtain lower respiratory tract specimens, the use of diagnostic tests with insufficient sensitivity, the absence of appropriate diagnostic testing methods, the undetectability of the virus at the time of the study, and the presence of unknown pathogens that were not identified. The low rate of virus detection among adults who were hospitalized for pneumonia highlights the need for more-sensitive diagnostic approaches, innovative discovery of pathogens, and assessing viruses in the past history (weeks before the presence of disease) [34]. Moreover, chronic obstructive pulmonary disease (COPD) exacerbation is a very important cause of ARIs and hospital admissions [35]. Only 7 of 16 studies included COPD in the etiologic data, and this information was unclear in the remaining studies, which might have underestimated the role of viral infection in these patients.

A previous etiological review focusing on young children aged <5 years [8] showed that RSV, Flu (including Flu A), PIV, hMPV, and RV were significantly more common in children hospitalized with acute lower respiratory tract infection than asymptomatic controls. The associations of these viruses (except RSV) with ARI and pneumonia were stronger among adults. This is in part because, in comparison to young children, the detection of viruses in the control group (ie, among individuals without respiratory symptoms or healthy controls) was less common in older adults, with the exception of RSV.

Several methodological issues could affect our results: sample size, age group, case ascertainment, clinical specimen, and diagnostic testing. Although a thorough search has been performed across 7 databases, including 3 Chinese-language databases, only 16 studies from the published literature were identified, which met our selection criteria. Not every virus of interest was tested in each study. The number of studies available was even smaller when subgroup analyses and sensitivity analyses were performed. Moreover, the sample size varied from 50 to 2320 adults in the case group and from 27 to 541 adults in the control group. The small sample size undoubtedly contributed to the imprecise 95% CIs around the ORs. Thus, we may have failed to detect clinically significant ARI-virus associations, owing to small sample sizes.

We aimed to stratify the association between common respiratory viruses in adults with ARI or pneumonia by age. However, most articles did not stratify and report data by age group. Instead, they summarized the result for the entire age group, usually in adults aged >18 years. Therefore, some of our meta-estimate ORs may not be representative of older adults who are aged ≥65 years. Since age might be a risk factor for ARI in adults (the rate of severe ARI increases as age advances), this could potentially affect the viral profile detected, introducing further heterogeneity [1].

Fifteen of 16 studies used passive clinic or hospital based case ascertainment. Among them, cases were recruited from inpatients, outpatients, emergency departments, or general practices, which might reflect different healthcare behavior and disease severity. Also, since the episodes of ARI and pneumonia were only diagnosed through routine care, this introduced bias, considering that testing was only done when the clinicians deemed it necessary to test. Similarly, 5 studies used community-based controls, while another 11 studies recruited older adult controls from hospitals or general practices. Hospital or clinical ascertained controls may not reflect the general population and may have other health conditions potentially affecting their viral carriage. Moreover, recruiting controls who were selected as healthy or without respiratory symptoms could favor those who were not exposed to the respiratory virus (yielding a falsely high OR). Therefore, we consider that the ideal control group for these studies would be a random sample of an age- and sex-matched population of older adults who are from the same area of residence and studied at the same time as cases.

All included studies obtained upper respiratory tract specimens (described as nasopharyngeal secretions). Assays might have specimen-specific sensitivities and specificities for detecting viruses, which could lead to heterogeneity in the estimation of virus-specific rates. The sensitivity of using nasopharyngeal washes for detecting any virus in adults was found to be higher than that for using nasopharyngeal swab specimens, which in turn was higher than that for using oropharyngeal swab specimens (84.9%, 73.3%, and 54.2%, respectively) [36]. The limited use (due to ethical concerns and feasibility) of invasive procedures to obtain lower respiratory tract specimens directly from the lung also influenced the diagnosis of viral infection in adults with ARI [37].

PCR and serology-based diagnostic testing are more sensitive for detecting respiratory viruses than other methods, such as antigen detection and culture. High sensitivity is important for accurate assessment of etiological contribution, particularly in older adults who may have a lower nasopharyngeal viral load and an atypical clinical presentation [7]. Moreover, despite being uncommon, detection of viral coinfection (range across studies, 1%–10%) may tend to overstate the contribution of individual respiratory viruses (although dual or multiple infections, in which both or several viruses have etiological importance, are possible). Bacterial coinfections (range across studies, 15%–26%) were also reported. With improving diagnostic methods, multiple etiological agents are increasingly identified simultaneously in older adults with ARI, making the individual contribution of each agent difficult to define.

Viruses can be detected in individuals with no respiratory symptoms. This is often seen in volunteer challenge studies and in some community surveys [38, 39]. The detection of viruses in control groups without respiratory symptoms might be due to a nascent infection or a persisting colonization from a previous infection [40]. These factors will tend to result in true associations being attenuated. The fact that molecular detection of viruses in older adults with ARI is higher than the detection rate in controls without respiratory symptoms may not necessarily indicate causation. Alternative explanations should be considered first before causality can be concluded. These include the respiratory viral infection acting as a so-called innocent bystander, without a causal role, and serving only as a predisposing risk factor for ARI. Similarly, the absence of a positive association (and AFE) does not mean that a virus is not a cause of ARI. Moreover, without establishing the temporal sequence of exposure and outcome, determinations of causality are less secure. Therefore, the association between viruses and ARI and pneumonia should be interpreted carefully.

In conclusion, this review provides clear evidence that is suggestive of the potentially causal role of RSV, Flu, PIV, hMPV, AdV, RV, and CoV in older adults with ARI and presents the first estimate of the proportion of ARI cases that can be attributed to virus exposure. Etiological studies, which simply report rates of viral identification as causal, should make attempts to interpret findings in terms of the proportion of ARI cases among older adults in whom a respiratory virus is identified that can be attributed to this viral exposure.

## STUDY GROUP MEMBERS

The RESCEU investigators are as follows: Harish Nair, Harry Campbell, Ting Shi, Shanshan Zhang, and You Li (University of Edinburgh); Peter Openshaw and Jadwicha Wedzicha (Imperial College London); Ann Falsey (University of Rochester); Mark Miller (National Institutes of Health–Fogarty); Philippe Beutels (Universiteit Antwerpen); Louis Bont (University Medical Center Utrecht); Andrew Pollard (University of Oxford); Eva Molero (Synapse); Federico Martinon-Torres (Servicio Galego de Saude); Terho Heikkinen (Turku University Central Hospital); Adam Meijer (National Institute for Public Health and the Environment); Thea Kølsen Fischer (Statens Serum Institut); Maarten van den Berge (Academisch Ziekenhuis Groningen); Carlo Giaquinto (PENTA Foundation); Rafael Mikolajczyk (Martin-Luther University Halle-Wittenberg); Judy Hackett and Eskinder Tafesse (AstraZeneca); Bing Cai and Charles Knirsch (Pfizer); Antonio Gonzalez Lopez, Ilse Dieussaert, Nadia Dermateau, and Sonia Stoszek (GlaxoSmithKline); Scott Gallichan, Alexia Kieffer, and Clarisse Demont (Sanofi Pasteur); Arnaud Cheret, Sandra Gavart, Jeroen Aerssens, Veronique Wyffels, and Matthias Cleenewerck (Janssen); and Robert Fuentes and Brian Rosen (Novavax).

## Supplementary Data

Supplementary materials are available at *The Journal of Infectious Diseases* online. Consisting of data provided by the authors to benefit the reader, the posted materials are not copyedited and are the sole responsibility of the authors, so questions or comments should be addressed to the corresponding author.

Supplementary Material

## Notes

***Acknowledgments.***  We thank Joris Menten from Janssen for reviewing the manuscript.

***Financial support.*** This work was supported by the Innovative Medicines Initiative 2 Joint Undertaking (grant 116019), which in turn receives support from the European Union’s Horizon 2020 Research and Innovation Programme and EFPIA.

***Potential conflicts of interest.*** A. R. F. reports grants from Janssen, Merck, Pfizer, and Gilead and personal fees from Sanofi Pasteur, outside the submitted work. H. C. reports grants from the European Union Innovative Medicines Initiative during the conduct of the study and grants and personal fees from the World Health Organization (WHO), Sanofi, and the Bill and Melinda Gates Foundation, all paid through the University of Edinburgh and outside the submitted work. H. N. reports grants from the Innovative Medicines Initiative during the conduct of the study; grants and personal fees from the WHO, Bill and Melinda Gates Foundation, and Sanofi; and grants from National Institute of Health Research, outside the submitted work. J. A. W. reports grants from GSK, Johnson and Johnson, GSK, Astra Zeneca, Boehringer Ingelheim, and Novartis and other support from Novartis, Boehringer Ingelheim, Astra Zeneca, and GSK, outside the submitted work. P. O. reports personal fees from a consultancy and the European Respiratory Society; grants from the Medical Research Council (MRC), the European Union, the National Institute for Health Research (NIHR) Biomedical Research Centre, the MRC and GSK, the Wellcome Trust, the NIHR (Health Protection Research Units), the NIHR (senior investigator support), and the MRC Global Challenge Research Fund; and nonfinancial support from AbbVie. P. O. is the elected President of the British Society for Immunology, which is an unpaid appointment but provides support for travel and accommodation at some meetings. All other authors report no potential conflicts.
