# Supplementary material for: The Etiological Role of Common Respiratory Viruses in Acute Respiratory Infections in Older Adults: A Systematic Review and Meta-analysis
Source: J Infect Dis. 2019 Mar 8:jiy662. doi: 10.1093/infdis/jiy662 (PMC7107439; doi:10.1093/infdis/jiy662)
Supplement: Supplementary Material [file jiy662_suppl_supplementary_material.docx]

Supplementary material for “The Etiological Role of Common Respiratory Viruses in Acute Respiratory Infections in Older Adults: A Systematic Review and Meta-analysis”

Search strategy

**Medline**

1. Prospective study.mp. or exp Prospective Studies/

2. control*.mp.

3. exp Case-Control Studies/ or case control.mp.

4. (case* adj2 control*).mp. [mp=title, abstract, original title, name of substance word, subject heading word, keyword heading word, protocol supplementary concept word, rare disease supplementary concept word, unique identifier]

5. virus.mp. or exp Viruses/

6. exp Virus Diseases/ or virus disease*.mp.

7. pneumonia.mp. or exp Pneumonia/ or exp Pneumonia, Viral/

8. bronchiolitis.mp. or exp Bronchiolitis/ or exp Bronchiolitis, Viral/

9. exp Respiratory Tract Infections/ or respiratory infection*.mp.

10. exp Respiratory Tract Diseases/ or respiratory disease*.mp.

11. 1 and 2

12. 3 or 4 or 11

13. 5 or 6

14. 7 or 8 or 9 or 10

15. 12 and 13 and 14

16. limit 15 to (humans and yr="1996 -Current" and ("all aged (65 and over)" or "aged (80 and over)"))

**Embase**

1. virus*.mp. or exp virus/

2. exp virus infection/ or virus disease*.mp.

3. exp community acquired pneumonia/ or exp pneumonia/ or exp virus pneumonia/ or exp infectious pneumonia/ or pneumonia.mp.

4. bronchiolitis.mp. or exp bronchiolitis/ or exp viral bronchiolitis/

5. exp respiratory tract infection/ or exp lower respiratory tract infection/ or respiratory infection*.mp.

6. exp respiratory tract disease/ or respiratory disease*.mp.

7. exp case control study/ or case control.mp.

8. (case* adj2 control*).mp. [mp=title, abstract, subject headings, heading word, drug trade name, original title, device manufacturer, drug manufacturer, device trade name, keyword]

9. prospective study.mp. or exp prospective study/

10. control*.mp. or exp control group/ or exp control/

11. 1 or 2

12. 3 or 4 or 5 or 6

13. 9 and 10

14. 7 or 8 or 13

15. 11 and 12 and 14

16. limit 15 to (human and yr="1996 -Current" and aged <65+ years>)

**Global Health**

1. virus.mp. or exp viruses/

2. viral disease*.mp. or viral diseases.sh.

3. exp community acquired pneumonia/ or pneumonia.mp. or exp pneumonia/

4. bronchiolitis.mp. or exp bronchiolitis/

5. respiratory infection*.mp.

6. respiratory disease*.mp. or respiratory diseases.sh.

7. case control.mp.

8. exp case-control studies/

9. (case* adj2 control*).mp. [mp=abstract, title, original title, broad terms, heading words]

10. prospective study.mp.

11. exp control/ or control*.mp.

12. 1 or 2

13. 3 or 4 or 5 or 6

14. 10 and 11

15. 7 or 8 or 9 or 14

16. 12 and 13 and 15

17. limit 16 to yr="1996 -Current"

**LILACS**

tw:(virus OR viral) AND tw:(pneumonia OR bronchiolitis OR alri OR lrti OR "lower respiratory" OR "respiratory infection") AND tw:(control)

**CNKI**

Topic: respiratory infection or pneumonia (vague)

And Topic: virus (vague)

And Topic: case control (vague)

And Topic: adult (vague)

Publication time: 1^st^ Jan 1996 – 10^th^ Aug 2017

**Wanfang Data**

All (vague): respiratory infection or

All (vague): pneumonia or

All (vague): virus and

All (vague): case control and

All (vague): adult

Time: 1996 – 2017

**Chongqing VIP**

Title/key word: respiratory infection

Or Title/key word: pneumonia

And Title/key word: virus

And Title/key word: case control

And Title/key word: adult

Time: 1996 – 2017

Supplementary Figure 1: Selection criteria

Studies in older adults aged ≥65 years;

Studies investigating acute respiratory infections (ARI), including pneumonia, as the primary outcome;

Studies where respiratory specimens were collected and respiratory virus identification was conducted using valid laboratory tests;

Case-control studies or prospective cohort studies that reported data in both case and control groups;

Studies reporting virus-specific proportions separately in both case and control groups;

Studies published between January 1^st^ 1996 – August 3^rd^ 2017 (August 10^th^ for Chinese databases);

Only studies where the case definition for ARI (or pneumonia) was clearly defined and consistently applied were included.

Supplementary Table 1: Definition of cases

|  | **Case definition** |
| --- | --- |
| Acute respiratory infection | Sudden onset of symptoms lasting less than 14 days;  At least one of the following: cough, sore throat, shortness of breath, runny rose;  Clinician’s judgement that illness is due to infection;  With or without fever. |
| Pneumonia | ARI cases with cough or difficult breathing and two of the following signs:  Breathing ≥20 breaths/min,  Night sweats,  Chest pain. |
| Severe or very severe pneumonia | Pneumonia and one or more of the following signs:  Breathing ≥30 breaths/min,  Fever ≥39°C,  Pulse 120 or more,  Lethargy,  Not able to walk unaided,  Uncomfortable lying down,  Severe chest pain,  Hypoxemia: SpO2 <90% on room air, or requirement for oxygen,  Mechanical ventilation. |

Supplementary Table 2: Characteristics of 16 included studies

| **Study** | **Age  Range** | **Specimen(s);**  **Diagnostic Test(s)** | **Bacteria  Tested** | **Case Group** | | **Control Group** | |
| --- | --- | --- | --- | --- | --- | --- | --- |
|  |  |  |  | **n; Ascertainment** | **Definition** | **n; Ascertainment** | **Definition** |
| Gothenburg, Sweden; U;  Oct (06-08) - Apr (06-08) [17] | 19 - 87 Y | NPS, throat swab;  mqPCR | Yes | 209; Passive (OP) | ARI | 100; Active (C) | Healthy (2W no RS or fever) |
| London, UK; U&R;  May 00 – Apr 01 [21] | 18 – 90 Y | Sputum, nasal aspirate; RT-PCR | Yes | 80; Passive (GP) | ALRI | 49; Passive (GP);  Matched (age, sex, season) | AS  (2M no RS) |
| Wageningen, Netherlands; U;  Oct 98 - Oct 99 [24] | ≥60 Y | Nose & throat swab, serum; PCR, serology | No | 107; Active (C) | ARI | 91; Active (C) | AS (8W no RS) |
| Christchurch, New Zealand; U;  Jul 99 - Jul 00 [23] | ≥18 Y | Throat swab;  PCR | Yes | 50; Passive (IP) | CAP | 50; Passive (IP);  Matched (age, sex) | AS |
| Beer-Sheva, Israel; U;  Nov (04-06) - Mar (04-06) [16] | ≥18 Y | OPS, NPS, NPW; qPCR | No | 183; Passive (IP) | CAP | 450; Passive (OP) | AS |
| Multicentre, USA; U;  Nov 11 - Jun 12 [20] | ≥18 Y | NPS, OPS; qPCR | No | 192; Passive (IP) | CAP | 238; Passive (OP) | AS  (2W no RS) |
| Taiwan, China; U;  Oct 12 - Jun 13 [19] | ≥18 Y | NPS, throat swab; PCR | No | 55; Passive (OP, ED) | ALRI | 27; Passive (OP) | AS |
| Istanbul, Turkey; U;  Jan - Dec 10 [18] | >16 Y | NPS; RT-PCR | Yes | 52; Passive (IP, OP) | P | 46; Passive (OP) | AS  (1M no RS) |
| Netherlands; U;  Oct 00 - Oct 03 [15] | Mean 35 Y | Nose, throat swab; viral culture, PCR | Yes | 376; Passive (GP) | ARI | 541; Passive (GP);  Matched (age) | AS  (2W no RS) |
| Guangzhou, China; U;  Apr - Dec 09 [22] | 35 – 77 Y | Throat swab;  PCR, cell culture | No | 149; Passive (IP) | CAP | 75; Active (C) | AS  (1M no RS) |
| Shenzhen,  China; U; Oct 07 - Oct 08 [25] | 16 – 89 Y | NPS; PCR | No | 192; Passive (IP) | CAP | 106; Active (C) | Healthy |
| Chengde, China; U;  96 – 09 [26] | 18 – 88 Y | NPA; PCR | No | 613; Passive (IP, OP) | ARI | 190; Active (C) | Healthy |
| Chicago and Nashville, US; U; Jan 10 - Jun 12 [1] | ≥18 Y | NPS, OPS; PCR | Yes | 2320; Passive (IP) | CAP | 238; Passive (OP) | AS |
| Sa Kaeo, Thailand; R; Sep 03 - Aug 05 [27] | ≥65 Y | NPS; RT-PCR | No | 531; Passive (IP) | P | 36; Passive (OP) | AS  (3D no RS) |
| Sa Kaeo, Thailand; R; Sep 04 - Aug 05 [28] | ≥65 Y | NPS; RT-PCR | No | 305; Passive (IP) | P | 36; Passive (OP) | AS  (3D no RS) |
| Rochester, USA; U; Jan - Apr  2004 [29] | 19-87 Y | Nasal swab; RT-PCR | No | 146; Passive (GP) | ARI | 146; Passive (GP);  Matched (age, sex) | AS  (1W no RS) |
| **Abbreviations**: U = Urban; R = Rural, NPA = Nasopharyngeal Aspirate; NPS = Nasopharyngeal Swab; NPW = Nasopharyngeal Wash; OPS = Oropharyngeal Swab; PCR = Polymerase chain reaction (m = multiplex; RT = reverse transcription; q = quantitative/real time); Active ascertainment: nurses evaluate the cases / controls during home visits (prospective community-based); Passive ascertainment: cases / controls are identified in hospitals / clinics; P = Pneumonia; ALRI = Acute Lower Respiratory Infection; ARI = Acute Respiratory Infection; CAP = Community Acquired Pneumonia; RS = Respiratory Symptoms; IP = Inpatient; OP= Outpatient; GP = General Practice; ED = Emergency Department; C = Community; D = Days; W = Weeks; M = Months; Y = Years; AS = Asymptomatic. | | | | | | | |

Supplementary Table 3: The meta analyses of the odds ratios (OR) and attributable fractions in the exposed (AFE) of each virus and its subtype within all included studies of ARI cases relative to asymptomatic controls

| **Virus** | | **Meta-Analysis** | | | **Subgroup analysis** | | | | | |
| --- | --- | --- | --- | --- | --- | --- | --- | --- | --- | --- |
|  |  |  |  |  | Developing countries | | | Industrialised countries | | |
|  |  | **n_s_** | **OR** (95% CI) | **AFE** (%) (95% CI) | **n_s_** | **OR** (95% CI) | **AFE** (%) (95% CI) | **n_s_** | **OR** (95% CI) | **AFE** (95% CI) |
| **RSV** | | 10 | **8.5** (3.9 to 18.5) | **88** (74 to 95) | 3 | **NA*** | **~100** | 7 | **8.5** (3.9 to 18.5) | **88** (74 to 95) |
|  | **A** | 1 | - | - | 1 | - | - | 0 | - | - |
|  | **B** | 0 | - | - | 0 | - | - | 0 | - | - |
| **Flu** | | 10 | **8.3** (4.4 to 15.9) | **88** (77 to 94) | 3 | **6.9** (2.1 to 23.1) | **85** (51 to 96) | 7 | **9.0** (4.2 to 19.2) | **89** (76 to 95) |
|  | **A** | 8 | **8.4** (3.9 to 17.8) | **88** (75 to 94) | 4 | **6.9** (2.1 to 23.1) | **85** (51 to 96) | 4 | **9.5** (3.6 to 24.9) | **89** (72 to 96) |
|  | **B** | 5 | **NA*** | **~100** | 2 | **NA*** | **~100** | 3 | **NA*** | **~100** |
|  | **C** | 0 | - | - | 0 | - | - | 1 | - | - |
| **PIV** | | 8 | **NA*** | **~100** | 2 | **NA*** | **~100** | 6 | **NA*** | **~100** |
|  | **1** | 2 | **NA*** | **~100** | 2 | **NA*** | **~100** | 0 | **-** | **-** |
|  | **2** | 0 | **-** | **-** | 0 | **-** | **-** | 0 | **-** | **-** |
|  | **3** | 3 | **NA*** | **~100** | 3 | **NA*** | **~100** | 0 | **-** | **-** |
|  | **4** | 0 | - | - | 0 | - | - | 0 | - | - |
| **hMPV** | | 7 | **9.8** (2.3 to 41.0) | **90** (57 to 98) | 1 | **-** | **-** | 6 | **9.8** (2.3 to 41.0) | **90** (57 to 98) |
| **AdV** | | 8 | **NA*** | **~100** | 3 | **NA*** | **~100** | 5 | **NA*** | **~100** |
| **RV** | | 11 | **7.1** (3.7 to 13.6) | **86** (73 to 93) | 3 | **8.7** (2.0 to 37.8) | **88** (50 to 97) | 8 | **7.0** (3.4 to 14.7) | **86** (70 to 93) |
| **BoV** | | 2 | **5.6** (1.3 to 23.7) | **82** (25 to 96) | 2 | **5.6** (1.3-23.7) | **82** (25 to 96) | 0 | **-** | **-** |
| **CoV** | | 8 | **2.8** (2.0 to 4.1) | **65** (49 to 76) | 1 | **-** | **-** | 7 | **2.9** (2.0 to 4.3) | **66** (50 to 77) |
|  | **HKU1** | 2 | **1.0** (0.0 to 52.9) | **2** (-4900 to 98) | 1 | **-** | **-** | 1 | **-** | **-** |
|  | **NL63** | 3 | **1.2** (0.3 to 5.0) | **19** (-223 to 80) | 1 | **-** | **-** | 2 | **1.2** (0.3 to 5.0) | **19** (-223 to 80) |
|  | **229E** | 2 | **6.3** (1.2 to 32.7) | **84** (17 to 97) | 1 | **-** | **-** | 1 | **-** | **-** |
|  | **OC43** | 4 | **4.2** (1.7 to 10.4) | **76** (42 to 90) | 2 | **NA*** | **~100** | 2 | **4.2** (1.7 to 10.4) | **76** (42 to 90) |
| Abbreviations: ns = Number of studies; NA = Not available; 95% CI = 95% Confidence interval; RSV = Respiratory syncytial virus; Flu = Influenza; PIV = Parainfluenza; hMPV = human metapneumovirus; AdV = Adenovirus; RV = Rhinovirus; BoV = Bocavirus; CoV = Coronavirus; OR = Odds ratio; AFE = Attributable fraction among the exposed.  * In all included studies for analysis, the virus was consistently identified in the case group but absent in the control group. This informs a strong association between the virus and ARI. Combining these studies into a meta-analysis would result in an extremely large meta-estimate OR. Therefore, the meta-analysis was not carried out. | | | | | | | | | | |

Supplementary Table 4: Sensitivity analyses to investigate the roles of viruses in adults hospitalised with ARI

| **Virus** | | **Meta-analysis (all)** | | | **Meta-analysis (inpatients as case group)** | | |
| --- | --- | --- | --- | --- | --- | --- | --- |
|  |  | **n_s_** | **OR** (95% CI) | **AFE** (%) (95% CI) | **n_s_** | **OR** (95% CI) | **AFE** (%) (95% CI) |
| **RSV** | | 10 | **8.5** (3.9 to 18.5) | **88** (74 to 95) | 4 | **8.5** (2.7 to 26.5) | **88** (63 to 96) |
|  | **A** | 1 | - | - | 0 | - | - |
|  | **B** | 0 | - | - | 0 | - | - |
| **Flu** | | 10 | **8.3** (4.4 to 15.9) | **88** (77 to 94) | 5 | **8.0** (3.1 to 20.8) | **88** (68 to 95) |
|  | **A** | 8 | **8.4** (3.9 to 17.8) | **88** (75 to 94) | 3 | **8.0** (3.1 to 20.8) | **88** (68 to 95) |
|  | **B** | 5 | **NA*** | **~100** | 1 | **NA*** | **~100** |
|  | **C** | 0 | - | - | 0 | - | - |
| **PIV** | | 8 | **NA*** | **~100** | 3 | **NA*** | **~100** |
|  | **1** | 2 | **NA*** | **~100** | 2 | **NA*** | **~100** |
|  | **2** | 0 | **-** | **-** | 0 | **-** | **-** |
|  | **3** | 3 | **NA*** | **~100** | 2 | **NA*** | **~100** |
|  | **4** | 0 | - | - | 0 | - | - |
| **hMPV** | | 7 | **9.8** (2.3 to 41.0) | **90** (57 to 98) | 3 | **9.8** (2.3 to 41.0) | **90** (57 to 98) |
| **AdV** | | 8 | **NA*** | **~100** | 5 | **NA*** | **~100** |
| **RV** | | 11 | **7.1** (3.7 to 13.6) | **86** (73 to 93) | 6 | **7.9** (3.5 to 18.0) | **87** (71 to 94) |
| **BoV** | | 2 | **5.6** (1.3 to 23.7) | **82** (25 to 96) | 1 | **-** | **-** |
| **CoV** | | 8 | **2.8** (2.0 to 4.1) | **65** (49 to 76) | 4 | **3.4** (2.0 to 5.8) | **71** (50 to 83) |
|  | **HKU1** | 2 | **1.0** (0.0 to 52.9) | **2** (-4900 to 98) | 2 | **1.0** (0.0 to 52.9) | **2** (-4900 to 98) |
|  | **NL63** | 3 | **1.2** (0.3 to 5.0) | **19** (-223 to 80) | 2 | **1.2** (0.3 to 5.0) | **19** (-223 to 80) |
|  | **229E** | 2 | **6.3** (1.2 to 32.7) | **84** (17 to 97) | 2 | **6.3** (1.2 to 32.7) | **84** (17 to 97) |
|  | **OC43** | 4 | **4.2** (1.7 to 10.4) | **76** (42 to 90) | 3 | **4.2** (1.7 to 10.4) | **76** (42 to 90) |

**Abbreviations:** n_s_ = Number of studies; NA = Not available; 95% CI = 95% Confidence interval; RSV = Respiratory syncytial virus; Flu = Influenza; PIV = Parainfluenza; hMPV = human metapneumovirus; AdV = Adenovirus; RV = Rhinovirus; BoV = Bocavirus; CoV = Coronavirus; OR = Odds ratio; AFE = Attributable fraction among the exposed.

***** In all included studies for analysis, the virus was consistently identified in the case group but absent in the control group. This informs a strong association between the virus and ARI. Combining these studies into a meta-analysis would result in an extremely large meta-estimate OR. Therefore, the meta-analysis was not carried out.
